# IMPACT OF GROUP-BASED DIGITAL GAMING INTERVENTION AMONG INDIVIDUALS LIVING WITH EARLY TO MODERATE DEMENTIA

**DOI:** 10.1093/geroni/igae098.0930

**Published:** 2024-12-31

**Authors:** Megumi Inoue, Michelle Hand, Naoru Koizumi, Limei Chen, Emma Booker

**Affiliations:** George Mason University, Fairfax, Virginia, United States; George Mason University, Fairfax, Virginia, United States; George Mason University, Fairfax, Virginia, United States; George Mason University, Fairfax, Virginia, United States; George Mason University, Fairfax, Virginia, United States

## Abstract

Slowing the progression of dementia and maintaining good behavioral and psychological symptoms are important aspects of life for individuals living with dementia and their caregivers. This study involved a group-based digital gaming system called Obie Technology as an interactive intervention for individuals living with dementia at a local adult day health center for 20 weeks, from mid-November 2023 to mid-April 2024. This gaming system promotes movement, stimulates cognitive activity, and encourages socialization. For example, colorful balloons float around the board or floor as clients try to hit as many as they can. A mixed methods approach with a pre-post design was employed to examine the effects of the intervention on cognitive function, mood, and behaviors in 24 individuals with early or moderate-level dementia. The average scores of the Montreal Cognitive Assessment (MoCA) at the baseline and midpoint (after 10 weeks) were 15.65 and 15.60, respectively, indicating no significant progression of dementia between these time periods. Participants’ depression level and neuropsychiatric symptoms presentation assessed by standardized instruments also showed no significant changes. The results from qualitative observations demonstrate that the group of people living with early-stage dementia in this study were more engaged and enjoyed the intervention compared to a group with moderate-stage dementia. They often comment that the intervention was fun, interesting, and enjoyable while actively engaging in the game with enhanced movement. In this presentation, we will present the findings based on the completed data, including the post-assessments.

